# Antibiotic Prescribing for Oro-Facial Infections in the Paediatric Outpatient: A Review

**DOI:** 10.3390/antibiotics7020038

**Published:** 2018-04-25

**Authors:** Najla Dar-Odeh, Hani T. Fadel, Shaden Abu-Hammad, Rua’a Abdeljawad, Osama A. Abu-Hammad

**Affiliations:** 1 Dental College & Hospital, Taibah University, Al Madinah Al Munawwarah 42353, Saudi Arabia; hfadel@taibahu.edu.sa (H.T.F.); oabuhammad@taibahu.edu.sa (O.A.A.-H.); 2 Faculty of Dentistry, University of Jordan, Amman 11942, Jordan; s.abuhammad@yahoo.com; 3 Department of Pediatrics, Ibn Alhaitham Hospital, Amman 11942, Jordan; ruaa.abdeljawad@hotmail.com

**Keywords:** antibiotics, prescribing, paediatric, orofacial infections, antimicrobial resistance

## Abstract

There are many reports on the complications associated with antibiotics abuse during the treatment of paediatric patients, particularly those related to antimicrobial resistance. The dental profession is no exception; there is growing evidence that dental practitioners are misusing antibiotics in the treatment of their paediatric patients. This review is directed to dental practitioners who provide oral healthcare to children. It is also directed to medical practitioners, particularly those working in emergency departments and encountering children with acute orofacial infections. A systematic search of literature was conducted to explore the clinical indications and recommended antibiotic regimens for orofacial infections in paediatric outpatients. The main indications included cellulitis, aggressive periodontitis, necrotizing ulcerative gingivitis, and pericoronitis. Amoxicillin was found to be the most commonly recommended antibiotic for short durations of 3–5 days, with metronidazole or azithromycin being the alternative antibiotics in penicillin-sensitive patients.

## 1. Introduction

Antibiotics continue to be the most commonly prescribed drugs in children and adults [1]. In England, for instance, it is estimated that 66.4% of dental prescriptions are antibacterial drugs [2]. Variable rates of antibiotic prescribing among European countries are attributed to cultural and social factors, in addition to variable levels of awareness about the problem of antibiotic resistance among healthcare providers [3]. Within the dental community, awareness of clinical indications of antibiotic prescriptions to the child dental patient is lacking. It was found that a substantial proportion of children who received dental treatment for pain or localized swelling under general anaesthesia had also received antibiotics, with wide variation in antibiotic regimens [4]. Further, among members of the American Academy of Pediatric Dentistry, there was a trend toward overuse of antibiotics for non-indicated clinical conditions, like pain relief, irreversible pulpitis, and localised dentoalveolar abscess [5]. Interestingly, the same study found that certain non-clinical factors initiated antibiotic prescribing for some clinicians, such as unavailability of close appointments and seeking parental satisfaction [5]. A similar trend was observed in developing countries, where a substantial proportion of dentists prescribe for non-indicated clinical conditions, such as dry socket, localised periapical infection, marginal gingivitis, periodontitis, and pulpitis [6]. This insufficient knowledge of the appropriate clinical indications is paralleled by lack of awareness of important interventions that promote the optimal use of antibiotics, such as antibiotic stewardship programs [5]. Other forms of abuse in prescribing antibiotics include prescribing broad-spectrum antibiotics for infections that can be treated by narrow-spectrum antibiotics, prescribing antibiotics for long periods, and adopting inappropriate dosing regimens.

All of the above factors might contribute to the emergence of antibiotic resistance among children. Children as young as 4 years were found to harbour multidrug resistant bacteria in their oral cavities [7,8]. Further, patients who received frequent prescriptions of amoxicillin, a common antibiotic in dental prescriptions [9], also exhibited a higher rate of amoxicillin-resistant oral streptococci [10]. In addition to the problem of antibiotic resistance, there are other complications associated with antibiotic prescribing in paediatric population. A significant proportion of practitioners prescribe mostly sugar-containing formulations at frequencies inconsistent with manufacturers’ recommendations, and for prolonged periods of time, that may reach 10 days [11]. The risk of developing diabetes in children due to sugar-containing medications cannot be overlooked. Moreover, generally, there is risk of development of allergy and asthma in children treated with antibiotics [12,13]. Early-life exposure to antibiotics is also thought to change intestinal microbiota, with subsequent adverse long-term effects like obesity [13]. Other complications include superinfections with *Candida* species [14] and photosensitivity [15,16]. Children are also at risk of gastrointestinal disturbances, like diarrhoea, which is generally more frequent with three-times-daily than twice-daily regimens [17]. Furthermore, exposure to amoxicillin during early infancy may be linked to developmental enamel defects on both permanent first molars and maxillary central incisors [18].

Consequently, an urgent need arises to create more concrete and definitive guidelines for dental antibiotic prescribing in children [19], for all those involved in the management of orofacial infections in children.

This review aims at:

1. Highlighting clinical indications of therapeutic antibiotic prescribing for orofacial infections in the paediatric outpatients;
2. Presenting recommended antibiotic regimens for each clinical indication.

## 2. Materials and Methods

### 2.1. Eligibility Criteria

We used the following inclusion criteria for this review:

1. Papers published in English;
2. Papers published in the past 20 years (from January 1998 to December 2017);
3. Clinical trials;
4. Case reports and series;
5. Reviews;
6. Expert opinions;
7. Clinical guidelines;
8. Patients: paediatric outpatients having orofacial infections (odontogenic infections, periodontal infections, pericoronitis);
9. Intervention: prescribing regimen of antibiotics including: name, dose and duration.

We used the following exclusion criteria:

1. In vitro and animal studies;
2. Neonatal orofacial infections that need hospitalization;
3. Paediatric dental in-patients;
4. Prophylactic antibiotic prescribing.

### 2.2. Search Methodology

A literature search was conducted in MEDLINE/PubMed, Web of Science, and Google Scholar databases using the following four combinations: odontogenic OR dental AND children AND antibiotic; aggressive AND periodontitis AND antibiotics; pericoronitis AND children AND antibiotics; necrotizing AND gingivitis AND children. The search was conducted in the period up to and including December 2017. Furthermore, reference lists of included articles were searched for suitable references.

### 2.3. Selection Strategy

Titles and abstracts of papers were independently screened by two reviewers (Najla Dar-Odeh and Osama Abu-Hammad). If keywords and other eligibility criteria were in titles and/or abstracts, the papers were selected for full text review. Full text was critically reviewed by the same authors and papers that fulfilled all of the selection criteria were processed for data extraction. The reference lists of all selected studies were hand searched for additional relevant articles. Disagreements between the two reviewers were resolved by discussion.

### 2.4. Assessment of Risk of Bias

Risk of bias was assessed based on criteria for judging risk of bias according to Cochrane Handbook for systematic reviews of interventions [20].

### 2.5. Data Synthesis

A narrative (descriptive) synthesis of data was employed in this review, due to heterogeneity of studies. Studies were categorized according to level of evidence and the type of infection addressed in these studies. The American Academy of Pediatric Dentistry—Useful Medications for Oral Conditions [21] was further consulted for citing the antibiotic doses based on weight.

## 3. Results

### 3.1. Search and Selection

First search on facial cellulitis of odontogenic origin, identified 1124 papers. Duplicate papers and those older than 1998 were removed, producing a total of 665 papers. After initial screening of titles and abstracts, another 642 papers were excluded, as these were on:

- Prophylactic antibiotic prescribing;
- Children with chronic diseases like HIV and cardiovascular disease;
- Older guidelines when new guidelines are present;
- Non-odontogenic conditions;
- Topical use of antibiotics;
- Antimicrobials other than antibiotics;
- A recommended antibiotic regime was not mentioned.

After full text reading two references were included (Table 1).

The second search on aggressive periodontitis in children produced a total number of 410 articles. After removal of duplicates and studies older than 1998, a total of 337 remained. After initial screening of titles and abstracts, 317 articles were excluded, leaving 20 articles based on the following exclusion criteria:

- Adult studies;
- Non-bacterial periodontal infections;
- Topical antibiotic therapy;
- Aggressive periodontitis associated with systemic diseases;
- Antimicrobials other than antibiotics.

After full text reading, 6 were included (Table 1).

The third search was on pericoronitis in children. A total of 8 studies were retrieved, 7 of them were studies for adults and children with systemic disease, and so only one study was included (Table 1).

A fourth search was on necrotizing gingivitis in children. A total of 150 studies were retrieved. After removal of studies older than 1998, 77 remained. Studies which included systemic diseases, adult patients, microbiological studies, epidemiological studies, infections of non-bacterial origin (*N* = 63) were excluded, and 14 studies remained. After full text reading, only one study was included (Table 1).

### 3.2. Clinical Indications and Recommendations for Paediatric Dental Antibiotic Prescribing

**Figure 1:**
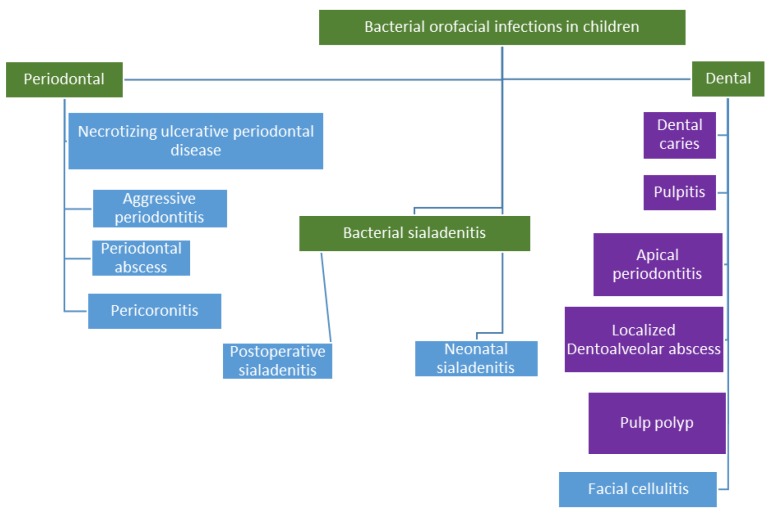
Orofacial infections in children. Infections quoted in blue boxes are best treated by operative intervention to remove the focus of infection in addition to adjunctive antibiotic therapy.

Children are susceptible to a number of bacterial infections in the orofacial region, and these infections are similar in most cases to adults. Figure 1 shows these orofacial bacterial infections in children with a particular focus on dental and periodontal infections.

Although all the aforementioned infections are bacterial in origin, only a limited number require therapeutic antibiotic prescribing in paediatric dental patients, since most of these infections respond very well to operative treatment by removing the source of infection, e.g., drainage of a dentoalveolar abscess, extraction of carious tooth, or pulp therapy for teeth with necrotic pulp, pulpitis, pulp polyp, and apical periodontitis, and scaling with removal of calculus in patients with periodontitis. When antibiotics are needed, they should not be used as the only line of treatment, but rather, as an adjunct to the operative therapy [22]. These infections will be discussed below, with particular emphasis on infections that require antibiotic therapy.

#### 3.2.1. Dentoalveolar Infections

Most orofacial infections are odontogenic in origin. The most common of these is dental caries and its complications (i.e., pulpitis, pulp necrosis, apical periodontitis, and periapical abscess). Children in many countries have high prevalence of caries, which can get to as high as 66% [30]. In his review, Finucane (2012) stresses that childhood caries (caries affecting children younger than 6 years) has to be treated, as it might be associated with pain, space loss, failure to thrive, and disruption to quality of life, among others [31]. Delay in treatment of dental decay will allow the infection to progress to cause infection of the tooth pulp (pulpitis); a bacterial infection that is associated with a more severe type of pain than dental caries. Untreated pulpitis may progress to pulpal necrosis, periapical abscess, or a dentoalveolar abscess, the latter being localised in the gingiva of the affected tooth. Chronic periapical abscess may sometimes drain in the sulcus by forming a sinus tract, and this is manifested clinically as a parulis. Another consequence of periapical infection is the formation of pulp polyp, which is a chronic hyperplastic lesion forming in the pulp chamber due to long-standing infection. Sinus tract, parulis, and pulp polyp are considered localised inflammatory lesions that are treated by operative intervention and not by antibiotic therapy. Localised infections in the form of caries, pulpitis, or periapical abscesses, are typically treated by operative intervention, either by pulp therapy, abscess drainage, or extraction, depending on the restorability of the tooth and the stage of development of the successor permanent tooth.

Sepsis is a serious complication of carious primary teeth, and this can progress to cellulitis. A substantial proportion of facial cellulites can be attributed to odontogenic infections [32]. If not properly treated, cellulitis can spread to the floor of mouth, leading to Ludwig’s angina, compromising the airway [32], or it can lead to blindness and involvement of mediastinum and spinal column [33,34,35]. Early treatment of odontogenic infections can prevent these morbidities. When infection becomes severe, the need for antibiotic administration may arise. Therefore, it is important to treat carious lesions in primary dentition, even if they become temporarily asymptomatic after pulpal necrosis. Dental anxiety—or fear from dental chair—in children has a prevalence that can get to as high as 39% [36], and is an important factor contributing to delayed treatment of localized odontogenic infections. Delayed treatment may also be related to parents’ dental fear [37], which may further complicate the problem.

When antibiotics are indicated for the treatment of an odontogenic infection, empiric therapy with a broad-spectrum antibiotic is recommended. For abscesses larger than 5 cm, cellulitis or conditions with mixed abscess-cellulitis, drainage and administration of antibiotics are indicated [38]. Some studies have recommended the use of amoxicillin and metronidazole, or amoxicillin with clavulanic acid, as an empiric treatment for odontogenic infections. However, a recent report showed a reduction in susceptibility of oral streptococci to amoxicillin [39]. Holmes and Pelleschia (2016) recommend amoxicillin, clindamycin, and azithromycin as empiric antibiotics for odontogenic infections [40]. Amoxicillin has good characteristics to be the drug of choice for orofacial infections; it is readily absorbed, it can be taken with food, and it is capable of resisting damage caused by gastric acidity [41]. In patients allergic to penicillin, researchers recommend clindamycin, azithromycin, metronidazole and moxifloxacin [40]. The latter has been found to be effective in severe odontogenic infections [42]. As for the duration of therapy, it is recommended to use short courses, particularly in children, to overcome the problem of compliance. In adults, the antibiotic regime of 1 g amoxicillin for 3 days was reported to be as clinically effective as long courses extending up to 7 days [39]. In children, the use of 2–3 day courses is recommended. This is supported by the findings that low daily dose or long duration of treatment with an oral beta-lactam (more than 5 days) can contribute to the appearance of penicillin-resistant pneumococci [43]. Recommended antibiotic regimens for odontogenic infections in children are explained in Table 1.

#### 3.2.2. Periodontal Diseases

There is growing evidence on the link of periodontitis and important systemic diseases, like cardiovascular disease and diabetes [44,45]. The problem is complicated by the high prevalence of periodontitis in the community with noticeable predilection to adult populations; as it is estimated to affect 85% of those older than 65 years of age [46]. The paediatric population are also susceptible to periodontal diseases, and these can be classified into [47]:

1. Plaque-induced periodontal diseases;
2. Aggressive periodontal disease;
3. Periodontal disease as a manifestation of systemic diseases;
4. Necrotizing periodontal diseases;
5. Abscesses of the periodontium;
6. Periodontal disease associated with endodontic lesions;
7. Developmental or acquired periodontal deformities and conditions.

The most common of the abovementioned conditions are related to bacterial plaque and poor oral hygiene, hence, the appropriate treatment is the operative intervention by scaling and oral hygiene measures to achieve mechanical removal of plaque and calculus. Plaque-induced gingivitis, i.e., reversible bleeding from the gingiva with no loss of periodontal attachment, demonstrated a prevalence among children and adolescents between 36% and 97% [48]. Periodontitis, which involves irreversible loss of attachment of mild, moderate, or severe intensities, can generally present in children in the following main forms [47]:

1. Chronic periodontitis;
2. Aggressive periodontitis;
3. Necrotizing ulcerative periodontitis;
4. Periodontitis associated with systemic diseases

Chronic periodontitis is closely associated with bacterial plaque, and occurs almost exclusively in adults [49]. It may also be observed in childhood or adolescence [49], although severe attachment loss in such a young age suggests a more aggressive form of periodontal disease [50]. Chronic periodontitis can be treated successfully via mechanical means, deeming the routine use of antimicrobials and antibiotics during treatment of questionable benefit, as opposed to the undeniable risks [51]. Aggressive periodontitis and necrotizing ulcerative periodontitis, on the other hand, are more characteristic entities that significantly benefit from adjunctive antibiotic therapy, in addition to operative intervention [52]. Management of periodontitis associated with systemic diseases broadly involves cause-related periodontal therapy through effective plaque removal and the control of the underlying systemic condition [52]. Whether this includes the adjunctive use of systemic antibiotics is dependent on a number of factors related to the specific condition, the host defences, and the physician’s opinion [52]. In this review, two types of periodontal diseases affecting children were considered: aggressive periodontitis and necrotizing ulcerative periodontal disease. These diseases may be encountered in the paediatric outpatient community as localised entities that require antibiotic therapy

##### Aggressive Periodontitis

Aggressive periodontitis, previously known as juvenile periodontitis, develops relatively early in life, and is characterised by rapid periodontal destruction [53]. Other possible features include possible familial aggregation, inconsistent low visible plaque deposits with advanced periodontal destruction, and elevated levels of certain specific microbial species [54]. When compared to other forms of periodontal diseases, aggressive periodontitis has a relatively low prevalence in populations. A recent study found that the prevalence among school children aged 13–19 is estimated to be 3.4% [55]. However, the importance of these diseases stems from their significant impact on patients’ quality of life, as they are characterised by a relatively rapid course of tissue destruction, with which timely intervention is crucial [53].

Aggressive periodontitis is best described as being multifactorial in nature, where genetic, microbiologic, immunologic, and environmental factors contribute to the initiation and progression of the disease [56]. The ideal treatment plan for managing aggressive periodontitis constitutes early diagnosis, mechanical, and chemical periodontal treatment, coupled with adjunctive antibiotic therapy, and long-term follow-up [25]. Mechanical treatment may involve nonsurgical as well as surgical procedures. Adjunctive use of systemic antibiotics in the treatment of aggressive periodontitis has yielded significant benefits [57]. The rational for using antibiotics in this context originates from the fact that aggressive forms of periodontitis represent a notable threat to oral and systemic health, making the prudent use of effective antibiotics ethically permissible in appropriately selected cases [58].

The most commonly used antibiotics for aggressive periodontitis include tetracyclines, amoxicillin, metronidazole, macrolides (spiramycin, erythromycin, and azithromycin), clindamycin, and ciprofloxacin, with the most common antibiotic combination regimen being metronidazole and amoxicillin [59]. Azithromycin (500 mg once daily for 3 days) has also been recommended [24]. Recommended antibiotic regimens are explained in Table 1.

While there is no consensus on the ideal regimen, it is important to prescribe an antibiotic in sufficient dose for adequate duration [59]. Another important clinical question is when it is best to start administering antibiotics in relation to the mechanical phase of treatment. Indirect evidence suggests that antibiotic intake should start on the day debridement is completed, and debridement should be completed within a short period of time, of less than one week [60].

##### Necrotizing Periodontal Lesions

Necrotizing periodontal diseases are a group of infectious diseases that include necrotizing ulcerative gingivitis, necrotizing ulcerative periodontitis, and necrotizing stomatitis. These diseases share common clinical features consisting of an acute inflammatory process and the presence of periodontal destruction [61]. In developing countries, the reported prevalence of necrotizing periodontal diseases is higher than that of developed countries, especially in children [62]. In South Africa, 73% of patients with necrotizing gingivitis were 5–12 years of age [63]. It affects predominantly young children in undeveloped countries, particularly in sub-Saharan Africa, and is caused by a mixed anaerobic bacterial infection in subjects with pre-existing debilitating conditions, mainly malnutrition, and less frequently, malaria, measles, and/or AIDS [64].

Clinically, necrotizing ulcerative gingivitis is characterized by the appearance of necrotic ulcers of the interdental papillae, associated with pain and halitosis. Necrotizing periodontal diseases can progress rapidly, causing severe tissue destruction. Accordingly, necrotizing gingivitis which is confined to the gingiva, can progress to necrotizing periodontitis to involve the alveolar bone. It is therefore important for these conditions to be managed promptly. Treatment includes gentle removal of hard and soft deposits, and the use of antiseptic mouth washes and oxygen–releasing agents [61]. In cases that show unsatisfactory response to debridement or if systemic signs and symptoms (fever and/or malaise) appear, the use of systemic antimicrobials may be considered [61]. Metronidazole is the first drug of choice, because of its activity against strict anaerobes. However, for aggressive oral infections, metronidazole may be used in combination with amoxicillin (metronidazole 250 mg 3 times/day with amoxicillin 250–375 mg 3 times/day for 7–10 days) as shown in Table 1. Other systemic drugs have also been proposed with acceptable results, including penicillin, tetracyclines, clindamycin, or amoxicillin plus clavulanate [61].

### 3.3. Recommended Antibiotic Regimens

Recommended antibiotic regimens are presented in Table 2, according to the references cited in Table 1. Duration of antibiotic therapy varied between studies addressing aggressive periodontitis. The case reports [25,28] recommended a 15-day duration, whilst the clinical trials [26,27] recommended a 7-day duration.

### 3.4. Assessment of Risk of Bias

Only one randomised controlled trial was included in this review [24]. Risk of bias for this study was judged to be low. More details are presented in Table 3.

## 4. Discussion

In the pre-antibiotic era, odontogenic infections were challenging to the oral surgeon, who had to deal with the frequently associated septic complications and fatal consequences [65]. Historically, the introduction of antibiotics in dental practice had a strong impact on the successful outcome of some critical dental treatments. From the year 1940 onwards, antibiotics have allowed for performing more apicectomies [66], and during World War II, penicillin and other antibiotics were introduced into surgeries, making maxillofacial surgeries much safer [67]. Those (and other) achievements should not be overturned because of malpractice in antibiotic prescribing. Whilst it is important to understand factors influencing the practitioners’ attitudes towards antibiotic prescribing, it is also necessary to provide clear guidelines based on sound clinical knowledge. Unfortunately, there is scarcity of resources on dental antibiotic prescribing for children. As can be noticed by this review, there are no clinical trials on antibiotic prescribing for facial cellulitis and pericoronitis in children, in contrast to other infections that received better attention, like otitis media and pharyngitis [17]. Further, studies that addressed aggressive periodontitis have pooled children and adults in the same patient group, and recruited a small sample size, which provides a very low level of evidence. Another important aspect is improving access to guidelines, and making these guidelines available to improve practitioners’ knowledge and practices. The planning and preparation of these guidelines should take into consideration that antibiotic prescribing for children is different to adults in a number of aspects. Medication intake in children remains a challenge. However, no matter how effective medication regimens are, treatment outcomes would be compromised if children and parents do not follow instructions adequately. Children’s behaviour may be characterised by lack of adherence to the prescription itself, so it is important that adherence and parents’ cooperation is guaranteed, for successful treatment. Prescribing medications that can be given once or twice daily, will improve patient’s compliance to the treatment [68]. Sugar-containing medications are expected to increase the patients’ adherence. However, sugar increases susceptibility to dental decay, tooth erosion, and associated complications, such as pulpitis and dentoalveolar abscess, emphasizing the importance of performing optimum oral hygiene activities during antibiotic therapy and beyond. Children may also show lack of cooperation when receiving dental treatment. Operative interventions should remain the first line of treatment for management of dental/periodontal infections in children. However, these interventions are highly resisted by children especially when treatment entails performing local anaesthetic injections, extractions, and preparing cavities or root canals for fillings. The situation may get complicated when parents’ faulty beliefs and perceptions encourage children’s dental phobic attitudes. All the aforementioned factors may help initiate antibiotic prescriptions by dentists, particularly those who lack patience and training in dealing with difficult children. Other inappropriate clinical practices that must be avoided are antibiotic prescribing for viral infections. Although many childhood diseases, like primary herpetic gingivostomatitis and infectious mononucleosis, may present with oral and systemic manifestations, they are still viral infections that should be treated by palliative treatment, rather than antibiotics. Worried parents may sometimes complicate the problem by expecting antibiotics and putting pressure on dentists to meet their expectations. In addition, parents’ lack of understanding of the diagnosis might hinder children’s intake of medications [68].

For children with little compliance, antibiotics with a long half-life like azithromycin become useful, as they only need to be taken once daily for three days and are well tolerated in children. On the other hand, azithromycin is more expensive than amoxicillin and clindamycin [40]. Furthermore, care should be taken when prescribing azithromycin, as it may lead to growth of azithromycin-resistant bacteria [69], and it may be associated with pro-arrhythmic effects [70]. Amoxicillin, on the other hand, is considered safer, especially when less frequent doses are used. A recent study found that using once or twice daily doses of amoxicillin, with or without clavulanate, were comparable with three doses for the treatment of acute otitis media in children [71]. The applicability of this finding in the treatment of dental infections needs to be further explored.

It was reported that physicians are more likely than dentists to prescribe antibiotics for dental problems [72]. Indeed, orofacial infections in children may be considered a common domain between dentistry, otolaryngology, and paediatrics, among others. This fact should encourage practitioners of different specialties to embrace the concept of interprofessional education and practice, so that medical and dental practitioners gain more knowledge about the correct clinical indications for antibiotic prescriptions.

Choice of the antibiotic and the appropriate dosing regimen may be challenging to the dentist. Certain antibiotics should be avoided in children, like fluoroquinolones, which can lead to chondrotoxicity in growing cartilage [40], and tetracycline, which can cause discoloration of permanent teeth. Further, choosing amoxicillin-clavulanic acid should cover a broader spectrum than amoxicillin. However, the use of clavulanic acid in the paediatric population has been associated with gastrointestinal disturbances (diarrhoea), but these can be reduced by using the two-daily rather than the three-daily regimen [73].

This review focused on the paediatric dental outpatient, so infections that require hospitalization for the treatment, like suppurative sialadenitis, were excluded. However, practitioners should be aware of the clinical presentation of sialadenitis since it may mimic other orofacial infections. Viral sialadenitis and juvenile recurrent parotitis represent the most common salivary gland diseases in children and adolescents [74]. The literature revealed a few types that can be described as suppurative sialadenitis. This bacterial infection may be attributed to sialolithiasis, a rare disease in children [75]. Acute postoperative sialadentitis has been described as a rare entity occurring after general anaesthesia in children, or following prolonged neurosurgical procedures with extreme positioning of the head and neck [76]. Due to the severity of infection and high possibility of compromising the airway, these children are usually hospitalised, and usually, clindamycin is prescribed [76]. Others prefer ampicillin with a beta lactamase inhibitor, third generation cephalosporins, vancomycin, and fluoroquinolones [77].

### Recommendations

When prescribing to children, it is important to follow certain measures to improve treatment outcomes. It will be beneficial to educate caregivers on the uses and potential side effects of antibiotics if the correct dosing regimen and treatment duration were not followed. This will help guarantee adherence by both caregivers and children. Health care professionals prescribing to children whether they are dentists or physicians are advised to consider the following when prescribing:

- Proper diagnosis is mandatory to design an appropriate treatment plan. To achieve the accurate diagnosis, history collection and clinical examination should be appropriately performed. Adjunctive diagnostic tools, like radiographs, can be of benefit, and should be used when indicated;
- It may seem more suitable to prescribe analgesics to supplement operative treatment for patients in pain;
- In case antibiotics were prescribed, children should be followed up for a few days to evaluate response to treatment, and the development of unwanted side effects;
- Dosing regimens for children can generally be estimated from their weight in kilograms, or from age, using the formula ((age + 4) × 2) if the child’s weight is unknown. In any case, the dose should not exceed the maximum adult dose;
- Treatment of orofacial infections entails collaborative efforts from all practitioners involved in the child’s healthcare.

## Figures and Tables

**Table 1:** Recommended antibiotic regimes for indicated conditions in the paediatric dental outpatient.

Oral Infection	Author/s (Year)	Type of Study	Indicated Antibiotic Regime	Indicated Antibiotic Regime in Penicillin-Allergic Patients	Additional Measures	Comments	Quality of the Evidence
Acute odontogenic abscess associated with raised axillary temperature and diffuse swelling	Palmer (2006) [22]	Expert opinion	Amoxicillin (2–3 days, max 5 days): <12 months: 62.5 mg tds 1–5 years: 125 mg tds 6–12 years: 250 mg tds Phenoxymethyl penicillin (2–3 days, max 5 days): <12 months: 62.5 mg qds 1–5 years: 125 mg qds 6–12 years: 250 mg qds	Metronidazole (3 days): 1–3 years: 50 mg tds 3–7 years: 100 mg bid 7–10 years: 100 mg tds >10 years: 200 mg tds Erythromycin (2–3 days, max 5 days): 1 month–2 years: 125 mg qds 2–12 years: 250 mg qds Azithromycin (2–3 days): 6 months–3 years: 10 mg/kg od 3–7 years 200 mg od 8–11 years: 300 mg od 12–14 years: 400 mg od >14 years: 500 mg od	Remove cause Establish drainage Review 2–3 days	Author recommends the use of these antibiotics in descending order: amoxicillin, phenoxymethyl penicillin, metronidazole and lastly erythromycin.	Low ^1^
Cellulitis	SDCEP [23]	Clinical guidelines	Amoxicillin (5 days): 6 months–1 year: 62.5 mg tds 1–5 years: 125 mg tds 6–18 years: 250 mg tds OR Phenoxymethyl penicillin (5 days): 6 months–1 year: 62.5 mg qds 1–6 years: 125 mg qds 6–12 years: 250 mg qds 13–18 years: 500 mg qds	Metronidazole Tabs, or Oral Suspension for 5 days: 1–3 years: 50 mg tds 4–7 years: 100 mg bid 8–10 years: 100 mg tds 11–18 years: 200 mg tds OR Clarithromycin (7 days): 1–5 years: 125 g bid 6–12 years: 187.5 mg bid 13–18 years: 250 mg bid			Low ^1^
Generalized aggressive periodontitis and localized aggressive periodontitis	Haas et al. (2008) [24]	RCT	Azithromycin 500 mg coated tablet once daily for 3 days.		Phase 1 consisted of two sessions of supragingival scaling and oral hygiene instructions. At day 15, a clinical examination was performed, and phase 2 started consisting of nonsurgical periodontal therapy with subgingival hand scaling and root planing. Phase 2 was completed within a period of 14 days. The subjects were given azithromycin the first treatment session of phase 2.	Patients were ≥13 years; One year follow up significant improvement.	Very low ^2^
Localized aggressive periodontitis	Muppa et al. (2016) [25]	Case report	Amoxicillin (50 mg/kg/day) (body weight in three divided doses) AND metronidazole 30 mg/kg/day for 15 days.		Further topical application of metronidazole in chlorhexidine (Rexidin-M gel) base was advised for 2 weeks. Vitamin B complex syrup was also included.	Child was 5 years old; Regular checkups and motivation for oral hygiene were done for 1½ years.	Very low ^3^
Localized Aggressive periodontitis	Beliveau et al. (2012) [26]	Retrospective analysis of clinical trial	500 mg of amoxicillin and 250 mg of metronidazole three times per day tds for 7 days.		Oral hygiene is mandatory.	Antibiotics were administered immediately after mechanical debridement.	Very low ^2^
	Merchant et al. (2014) [27]	Clinical trial	Same as above			Dose modified for children less than 40 kg.	Very low ^4^
	Seremidi et al. (2012) [28]	Case report	Amoxycillin 50 mg/kg and metronidazole 30 mg/kg tds) for 2 weeks.		The oral health preventive program included oral hygiene instructions and more specifically toothbrushing twice daily with a fluoridated toothpaste, use of dental floss for interdental cleaning, and use of disclosing tablets to increase the effectiveness of plaque removal. Dietary instructions (decrease of sweets intake up to once per day) were also given. In office fluoride application was carried out every 3–4 mοnths. Prescription of 0.2% chlorohexidine mouthrinse for 10 days.	8-year-old boy; Antibiotics were also administered at the end of the second visit of periodontal therapy which included full mouth scaling and root planing under local analgesia in two visits within a one-week interval.	Very low ^3^
Ulcerative necrotizing periodontitis	SDCEP [23]	Clinical guidelines	3-day regimen Amoxicillin: 6 months–1 year: 62.5 mg tds 2–5 years: 125 mg tds 6–18 years: 250 mg tds	3-day regimen Metronidazole: 1–3 years: 50 mg tds 4–7 years: 100 mg bid 8–10 years: 100 mg tds 11–18 years: 200 mg td			Low ^1^
Pericoronitis	SDCEP [23]	Clinical guidelines	3-day regimen Amoxicillin: 6 months–1 year: 62.5 mg tds 2–5 years: 125 mg tds 6–18 years: 250 mg tds	3-day regimen Metronidazole: 1–3 years: 50 mg tds 4–7 years: 100 mg bid 8–10 years: 100 mg tds 11–18 years: 200 mg td			Low ^1^

RCT: randomized controlled trial; tds: three times daily; qds: four times daily; od: once daily; bid: twice daily. Quality of evidence: GRADE-Working Group [29]. ^1^ Expert opinion or clinical guidelines; ^2^ Total sample includes children and adults. Number of children was not stated; ^3^ Case report; ^4^ Small sample size (22 participants).

**Table 2:** Recommended antibiotic regimens for orofacial infections in children.

Infection	Recommended Antibiotic Regimen	Recommended Antibiotic Regimen for Penicillin-Allergic Patient
Cellulitis	Amoxicillin (2–3 days, max 5 days): Children >3 months and <40 kg: 20–40 mg/kg/day in divided doses 8 hourly OR 25–45 mg/kg/day in divided doses 12 hourly Children >40 kg: 250–500 mg 8 hourly OR 500–875 mg 12 hourly OR Phenoxymethyl penicillin: (2–3 days, max 5 days) Children <12 years: 25–50 mg/kg/day in divided doses 6 hourly (max 3 g/day) Children ≥12 years: 250–500 mg 6 hourly	Metronidazole (3 days): Children: 30/mg/kg/day in divided doses 6 hourly (max 4 g/24 h) Adolescents and adults: 7.5 mg/kg 6 hourly (max 4 g/24 h) OR Azithromycin: Children >6 months up to 16 years: 5–12 mg/kg daily for 3 days (max 500 mg/day) OR 30 mg/kg as a single dose (max 1500 mg) OR Clarithromycin (7 days): 7.5 mg/kg 12 hourly 13–18 years: 250 mg 12 hourly
Aggressive periodontitis	Amoxicillin (50 mg/kg/day) AND Metronidazole 30 mg/kg/day 8 hourly for 7 days	Azithromycin (3 days): 10 mg/kg daily OR Metronidazole: 30 mg/kg/day 8 hourly for 7 days
Necrotizing ulcerative gingivitis	Amoxicillin (3 days): Children >3 months and <40 kg: 20–40 mg/kg/day in divided doses 8 hourly OR 25–45 mg/kg/day in divided doses 12 hourly Children >40 kg: 250–500 mg 8 hourly OR 500–875 mg 12 hourly	Metronidazole (3 days): Children: 30/mg/kg/day in divided doses 6 hourly (max 4 g/24 h) Adolescents: 250 mg 6 hourly OR 500 mg 8 hourly
Pericoronitis	Amoxicillin (3 days): Children >3 months and <40 kg: 20–40 mg/kg/day in divided doses 8 hourly OR 25–45 mg/kg/day in divided doses 12 hourly Children >40 kg: 250–500 mg 8 hourly OR 500–875 mg 12 hourly	Metronidazole (3 days): Children: 30/mg/kg/day in divided doses 6 hourly (max 4 g/24 h) Adolescents: 250 mg 6 hourly OR 500 mg 8 hourly

**Table 3:** Assessment of risk of bias for Haas et al. (2008) [24].

Domain	Support for Judgment	Authors’ Judgment
**Selection bias**		
Random sequence generation	Participants were randomly assigned by means of a draw	Low risk of bias
Allocation concealment	Medications were stored in opaque-coloured bottles identified only by the respective code of each participant	Low risk of bias
**Performance bias**		
Blinding of participants	Participants were masked from medications types	Low risk of bias
Blinding of personnel	Both periodontists involved in the treatment and clinical examination were masked from the identity of participants	Low risk of bias
**Detection bias**		
Blinding of outcome assessor	Blinding was ensured	Low risk of bias
**Attrition bias**		
Incomplete outcome data	There was no drop out of participants	Low risk of bias
**Reporting bias**		
Selective reporting	The article includes all expected outcomes, including those that were pre-specified	Low risk of bias
